# A Machine Learning Approach for Early Identification of Prodromal Parkinson’s Disease

**DOI:** 10.7759/cureus.63240

**Published:** 2024-06-26

**Authors:** Anisha Vaish

**Affiliations:** 1 Neurology, Tesla STEM (Science, Technology, Engineering & Math) High School, Redmond, USA

**Keywords:** “healthcare tech contest”, parkinson's disease, prodromal, machine learning (ml), early identification and diagnosis

## Abstract

Parkinson's disease (PD) affects approximately 6 million people worldwide. Data analysis of early PD symptoms using machine learning (ML) models may provide an inexpensive, non-invasive, and simple method for the remote diagnosis of early PD. The aim of this project was to analyze voice, computer keystrokes, spiral drawings, and gait data involving PD patients and controls available in public databases using ML models and identify early PD characteristics that are more pronounced than others. An ML model was developed using Random Forest to analyze existing clinical data for PD patients, prodromal PD patients with REM (rapid eye movement) sleep behavior disorder (RBD) symptoms, and non-PD healthy controls. We reviewed and collected data from the UCI (University of California Irvine) Machine Learning Repository, PPMI (Parkinson’s Progression Markers Initiative), and Kaggle databases. ML analysis was carried out on voice samples in PD and RBD patients, computer keystroke data, spiral drawings, and gait datasets. The ML prediction model developed may be helpful in improving risk prediction for PD, enabling early intervention and resource prioritization. The ML study suggests that voice analysis is the most robust test, followed by computer keystroke data, spiral drawings, and gait analysis, in that order. Voice is affected even in RBD patients, revealing that it is a sensitive and early measure of prodromal PD. The low accuracy of the analysis indicates that several PD-positive samples may remain undetected and unclassified. Combining all four features, that is, voice analysis, computer keystroke data, spiral drawings, and gait analysis, may improve the overall accuracy.

## Introduction

Personalized medicine holds the promise of specialized treatment and management of diseases for specific patients. The success of personalized medicine depends on accurate, early, and cost-effective disease diagnosis. The availability of clinical, genomic, and demographic data, along with advances in machine learning (ML) and artificial intelligence (AI), has the potential for early disease diagnosis in a cost-effective and timely manner [1].

Parkinson’s disease (PD) is a debilitating condition that significantly impacts the quality of life for many years. PD is the second most common neurodegenerative disease, affecting approximately 6 million people worldwide and about 1.2 million in the United States (www.parkinson.org) [2]. The number of PD patients is expected to rise as the population over 60 years of age increases [3]. PD causes symptoms including tremors, soft speech, slowness of movement, muscle rigidity, and shuffling gait, resulting in falls and injuries [3]. Currently, there are no approved medications to slow disease progression. While medications are available for symptomatic treatment, treatment-resistant symptoms emerge as PD advances [4]. Consequently, PD poses a significant financial burden of $51.9 billion in the United States and emotional stress for patients, caregiving families, and the healthcare system [5,6].

The optimal time to introduce disease-modifying treatment in PD is during the prodromal phase [7]. Therefore, early diagnosis of PD is essential for better management and outcomes [8]. A recent study has attempted to develop a multi-modal ML approach for predicting PD [2].

The accepted diagnostic criteria for PD include the presence of bradykinesia and at least one other motor symptom such as rest tremor or cogwheel rigidity (https://brainbanknetwork.ac.uk). Bradykinesia refers to slow or difficult movement, and cogwheel rigidity is a type of rigidity in which muscle responds with a cogwheel-like “catch and give” in response to constant force while moving a joint [9]. When PD motor symptoms manifest, 50% of dopaminergic nerve cells are already lost, making it too late for neuroprotective treatments to slow progression; thus, treatment focuses on symptomatic control [10].

PD results from neurodegeneration that begins long before the manifestation of motor symptoms. The latent phase, called prodromal PD, includes symptoms such as REM (rapid eye movement) sleep behavior disorder (RBD), soft speech, decreased smell, and constipation. Premotor symptoms of PD can precede motor symptoms by years or decades; this period is referred to as the premotor phase [11]. Data analysis using ML models may provide a method for identifying signs indicative of early PD before obvious motor signs manifest, allowing an opportunity for early intervention and in the future initiation of neuroprotective therapies to slow the progression of this devastating disease [10].

Early identification of PD may provide the opportunity for timely intervention which can have a huge societal impact of improving the quality of life for people with PD and decreasing the financial impact on the healthcare system [4].

Vocal impairment, including dysphonia and dysarthria in PD patients, is one of the earliest indicators of the disease onset [12,13]. Approximately 90% of PD patients show some form of vocal impairment [14,15]. Because voice measurement is non-invasive and simple to carry out, it has drawn significant attention for detecting and tracking the progression of PD [16,17].

Computer keyboard typing information, including key hold time and latency, can be used to identify unique users [18,19]. PD affects finger movements, which can be assessed using computer keyboard tapping timing information [20]. A software application called Tappy can capture keystroke events (Tappy keystroke) such as hold time, latency, and flight time, providing an objective and accurate detection of PD, especially in the early stages when motor symptoms may not be observable [20].

Similar to typing, handwriting is another characteristic affected by PD and has been suggested as a reliable marker for PD diagnosis [21]. To assess the effect of PD on patients' handwriting, the Dynamic Spiral Test (DST) and Static Spiral Test (SST) have been developed and proposed for PD diagnosis [22]. Gait is also affected in PD patients due to a combination of the decline in executive functioning as well as motor impairment. Gait variability and rhythmicity can be affected early in PD, especially while dual or multitasking [23].

Problems with existing clinical biomarker studies include (1) small sample sizes, (2) different sample sizes of clinical versus healthy controls, (3) different variables studied in various samples, (4) lack of prospective studies analyzing changes in clinical parameters over time, (5) clinical parameters affected by conditions other than PD, and (6) lack of racial and socioeconomic heterogeneity, making the data difficult to generalize [2,20].

An ideal solution would be a unique combination of clinical biomarkers that are inexpensive, home-based, integrated into daily activities, and analyzable remotely, creating a clinical signature for early PD. The aim of this project is to analyze voice data, including that of patients with RBD since they are at high risk for PD, computer keystroke data, spiral drawings, and gait data in PD patients and controls available in public databases using ML models to identify pronounced PD characteristics. Patients with RBD, involving the acting out of dreams during the REM phase of sleep, are at extremely high risk (80%) of developing PD [24].

This project has the potential to identify PD characteristics useful for early identification in the prodromal phase and for telemonitoring patients in remote areas [25].

## Materials and methods

A Random Forest ML model was developed to analyze existing biomedical data for PD and non-PD patients (healthy controls). The data was analyzed using a Random Forest model because it can reduce high variance from a flexible model such as a decision tree by combining many trees into one ensemble model. Therefore, the model is less prone to overfitting. Additionally, the Random Forest model is less computationally expensive and does not require a graphics processing unit (GPU) to complete training.

The UCI (University of California Irvine) Machine Learning Repository, PPMI (Parkinson’s Progression Markers Initiative), and Kaggle databases were reviewed, and it was found that Kaggle datasets are much cleaner. Therefore, all voice, Tappy keystroke, spiral drawing, and gait datasets were obtained from the Kaggle database. The current study did not need IRB approval since only publicly available data was used.

After downloading the data CSV files from the Kaggle database, they were imported into the local drive. The Pandas, Seaborn, NumPy, and Matplotlib libraries, along with tools from scikit-learn (sk-learn), such as pipeline, column transformer, StandardScaler, OneHotEncoder, MinMaxScaler, KBinsDiscretizer, LabelEncoder, train_test_split, Stratified K-Fold, cross_validate, ensemble, tree, LogisticRegression, and KNeighborsClassifier metrics, were imported into a Jupyter Notebook, which is an open-source web application that supports creating and sharing documents containing text, live code, equations, and visualizations.

The CSV files were loaded into the Jupyter Notebook. The columns containing unique identifiers for PD patients and controls were removed. The data was analyzed to check if it was balanced between the number of healthy controls and PD patients. The data files were further formatted for processing. In each dataset, there were more PD patients compared to healthy controls. The distributions of the variables were examined, and numeric and categorical features were identified. Outliers were handled by ensuring all values were within the 1st and 99th percentiles.

**Figure 1:**
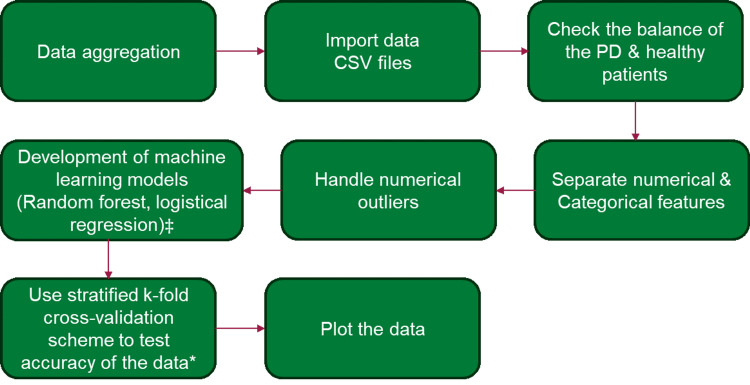
A summary of workflow and research procedures. ‡ Maintained approximately 80/20 ratio between training and test sets; * Maintaining as close as possible ratios of PD patients to healthy controls. The number of folds ranges from 3-5 across different data sets based upon how many pieces of data were available in the data set. PD: Parkinson's disease.

The data was divided into 80/20 training and testing sets, roughly maintaining the proportions of PD to control patients in the testing sets. An 80/20 split of training and testing sets were also maintained in all instances of Stratified K-Fold cross-validation to test the accuracy of our model. The model measured accuracy, precision, recall (sensitivity), and F1 score. Accuracy is the number of correct predictions over all predictions. Precision is a measure of how many of the positive predictions made are correct (i.e., true positives). Recall (sensitivity) is the measure of how many of the positive cases are correctly predicted over all the positive cases in the data. The F1 score combines precision and recall and measures the harmonic mean of the two values. A threshold value of 0.5 was maintained. A summary of the workflow and research procedures is shown in Figure 1.

Data sets

Voice Dataset

The voice data originally came from the UCI Machine Learning Repository [25]. The dataset was created at the University of Oxford in collaboration with the National Centre for Voice and Speech, Denver, Colorado, by recording speech signals. Feature extraction methods were employed for general voice disorders. The main features used in this study included PPE (pitch period entropy), F0 (the fundamental frequency or pitch of vocal oscillation), jitter (the extent of variation in speech F0 from vocal cycle to vocal cycle), shimmer (the extent of variation in speech amplitude from cycle to cycle), NHR (noise-to-harmonics ratio, the amplitude of noise relative to tonal components in the speech), RPDE (recurrence period density entropy), DFA (detrended fluctuation analysis), MDVP (Kay Pentax Multi-Dimensional Voice Program), HNR (harmonics-to-noise ratio), shimmer:DDA (average absolute difference between consecutive differences in the amplitudes of consecutive periods), shimmer:APQ3 (Three-point Amplitude Perturbation Quotient), shimmer:APQ5 (Five-point Amplitude Perturbation Quotient), MDVP:shimmer (Kay Pentax MDVP local shimmer), MDVP:PPQ (Kay Pentax MDVP Five-point Period Perturbation Quotient), MDVP:RAP (Kay Pentax MDVP Relative Amplitude Perturbation), MDVP:jitter(Abs) (Kay Pentax MDVP absolute jitter in microseconds), MDVP:jitter(%) (Kay Pentax MDVP jitter as a percentage), and jitter:DDS (average absolute difference of differences between cycles, divided by the average period) [25]. The D2 represents the correlation dimension calculated by first time-delay embedding the signal to recreate the phase space of the nonlinear dynamical system [25]. RPDE and DFA can detect general voice disorders [25]. PPE is a measure of changes in speech specific to PD [25]. MDVP allows comparison with other studies. The data was originally used for assessing the suitability of dysphonia measurements for telemonitoring of PD [25]. This dataset is composed of a range of biomedical voice measurements from 31 people, of which 23 were PD patients and the rest were controls. The data was arranged in a table for analysis. Each column in the table represents a particular voice measure, and each of the 195 rows corresponds to a voice recording from these individuals ("name" column). The analysis was conducted to identify healthy controls from PD patients. The "status" column refers to 0 for healthy controls and 1 for PD. The table was saved in ASCII CSV format. Approximately six recordings were conducted for each patient.

Tappy Keystroke Dataset 

The dataset contains naturalistic typing keystroke data from personal computers collected using the app, Tappy, from more than 200 subjects with PD and healthy controls over weeks or months. Tappy recorded the timing of key press and release events in milliseconds and the asymmetry between the right and left hands [20]. Key features recorded were hold time (the time between pressing and releasing each key); elapsed time (the time (ms) between releasing a key and pressing the subsequent key); keyside_L (keys pressed by the left hand); keyside_R (keys pressed by the right hand); keyside_S (space key because it can be pressed by either the left hand or the right hand); and next keyside (latency between successive keystrokes {elapsed time in ms from key down of one key until the next key down} categorized by same hand {LL or RR}, opposite hand {LR and RL}, and between the space key and left- or right-hand keys) [20].

Spiral Drawings Dataset

**Figure 2:**
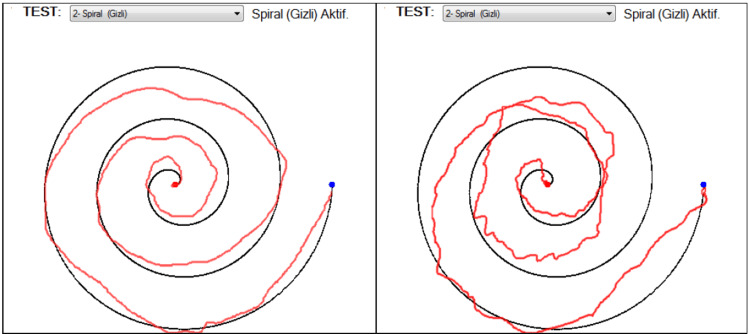
A representative DST drawing of a PD patient that scored highest (left) and lowest (right). DST: Dynamic Spiral Drawing Test; PD: Parkinson's disease.

The dataset contains spiral drawings from 62 people with PD and 15 healthy individuals [22]. Patients were asked to perform the Static Spiral Drawing Test (SST) and Dynamic Spiral Drawing Test (DST), where the spiral blinks on the computer screen, and the Stability Test on Certain Point (STCP), where subjects are asked to hold a digital pen on a red point without touching the screen. Controls have similar results on DST and SST, whereas PD patients perform worse on DST compared to SST. A representative DST drawing of a PD patient that scored highest (left) and lowest (right) is shown in Figure 2 [22].

*Gait Analysis Dataset* 

This database contains gait data from 93 patients with PD and 73 healthy controls. Subjects had 8 sensors underneath each foot which measured the vertical ground reaction force. This was calculated by measuring force in newtons as a function of time as they walked at a usual pace for approximately two minutes on flat ground [23,26,27].

Voice Analysis in Patients With RBD Dataset

Automated speech analysis was conducted in patients with RBD to identify alterations in natural speech in prodromal PD. The dataset includes 30 patients with newly diagnosed PD not yet on medications, 50 patients with RBD, and 50 healthy controls (HC) without any neurological or communication disorders. All subjects read standardized, phonetically-balanced text of 80 words and a monologue for approximately 90 seconds. Speech features were analyzed by an automated algorithm [28]. The main features analyzed in this study were RST (rate of speech timing), AST (acceleration of speech timing), DPI (duration of pause intervals), EST (entropy of speech timing), DUS (duration of unvoiced stops), DUF (decay of unvoiced fricatives), DVI (duration of voiced intervals), GVI (gaping in-between voiced intervals), RSR (rate of speech respiration), PIR (pause intervals per respiration), RLR (relative loudness of respiration), LRE (latency of respiratory exchange), RSR (rate of speech respiration), PIR (pause intervals per respiration), and RLR (relative loudness of respiration) [28].

## Results

Voice data analysis

**Figure 3:**
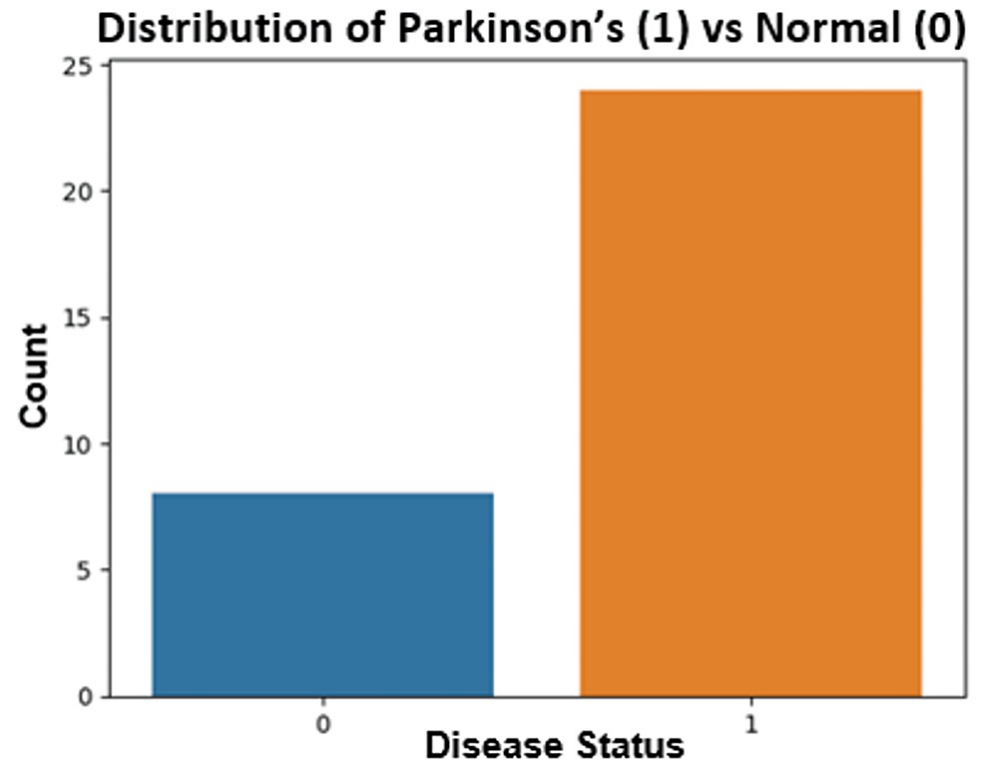
Distribution of the number of Parkinson’s disease (PD) patients versus controls in voice datasets. 0 represents control subjects, and 1 represents PD patients. "Count" represents the number of control subjects or PD patients.

The Random Forest analysis of voice data resulted in the highest accuracy, precision, recall (sensitivity), and F1 score. The analysis revealed an accuracy of 88.72%, a precision of 90.86%, a recall of 95.22%, and an F1 score of 92.77%. Figure 3 depicts the distributions of the number of PD patients versus controls in the voice datasets.

**Figure 4:**
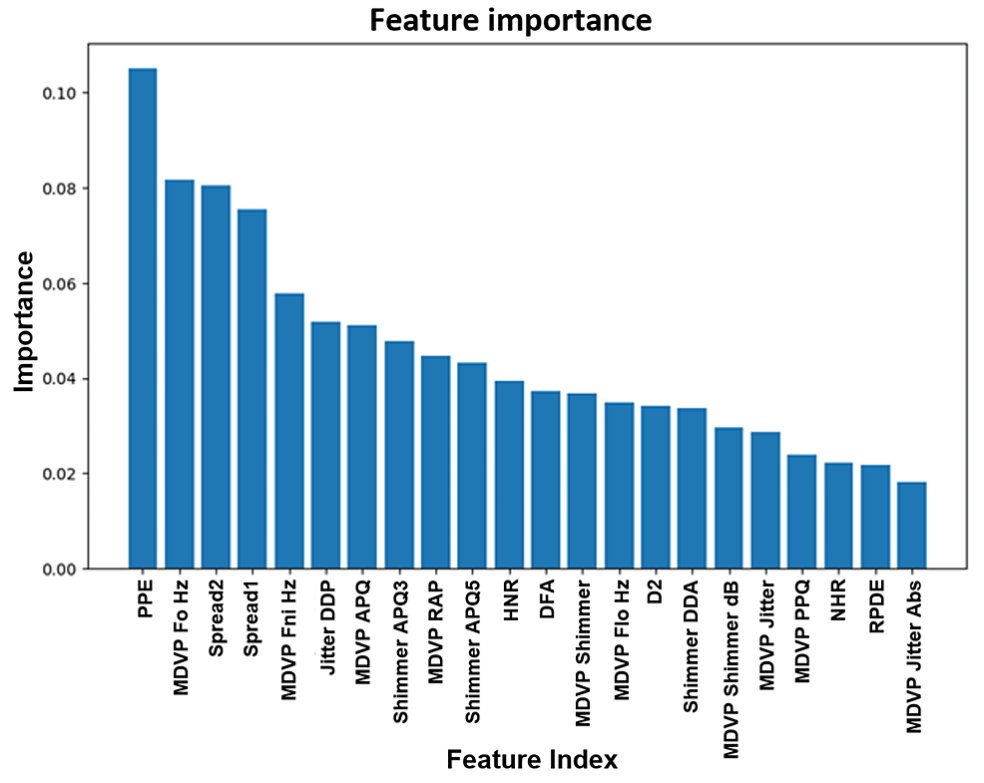
Feature importance in voice data set analysis. PPE: pitch period entropy; MDVP: Kay Pentax Multi-Dimensional Voice Program; F0: the fundamental frequency or pitch of vocal oscillation; jitter: the extent of variation in speech F0 from vocal cycle to vocal cycle; shimmer: the extent of variation in speech amplitude from cycle to cycle; NHR: noise-to-harmonics ratio, the amplitude of noise relative to tonal components in the speech; RPDE: recurrence period density entropy; DFA: detrended fluctuation analysis; HNR: harmonics-to-noise ratio; Shimmer DDA: shimmer:DDA, average absolute difference between consecutive differences in the amplitudes of consecutive periods; Shimmer APQ3: Shimmer:APQ3, Three-point Amplitude Perturbation Quotient; Shimmer APQ5: Shimmer:APQ5, Five-point Amplitude Perturbation Quotient; MDVP Shimmer: MDVP:Shimmer, Kay Pentax MDVP local shimmer; MDVP PPQ: MDVP:PPQ, Kay Pentax MDVP Five-point Period Perturbation Quotient; MDVP RAP: MDVP:RAP, Kay Pentax MDVP Relative Amplitude Perturbation; MDVP Jitter(Abs): MDVP:Jitter(Abs), Kay Pentax MDVP absolute jitter in microseconds; MDVP Jitter(%): MDVP:Jitter(%), Kay Pentax MDVP jitter as a percentage; Jitter DDS: Jitter:DDS, average absolute difference of differences between cycles, divided by the average period). “Spread” represents the spread of non-standard measures or comparison of traditional with non-standard measures.

Figure 4 depicts the feature importance in the voice dataset analysis. Feature importance is calculated by normalizing the features against the sum of all feature values in the Random Forest model tree and dividing it by the total number of trees in the model. When building a model based on voice data, important features to consider are PPE (pitch period entropy), ‘MDVP F0 Hz’ (Multi-Dimensional Voice Program), and spread. MDVP can extract 33 features/parameters from a voice sample and has the ability to provide rapid quantitative voice assessments. A model can be built from the MDVP data to detect early PD.

Tappy keystroke data analysis

**Figure 5:**
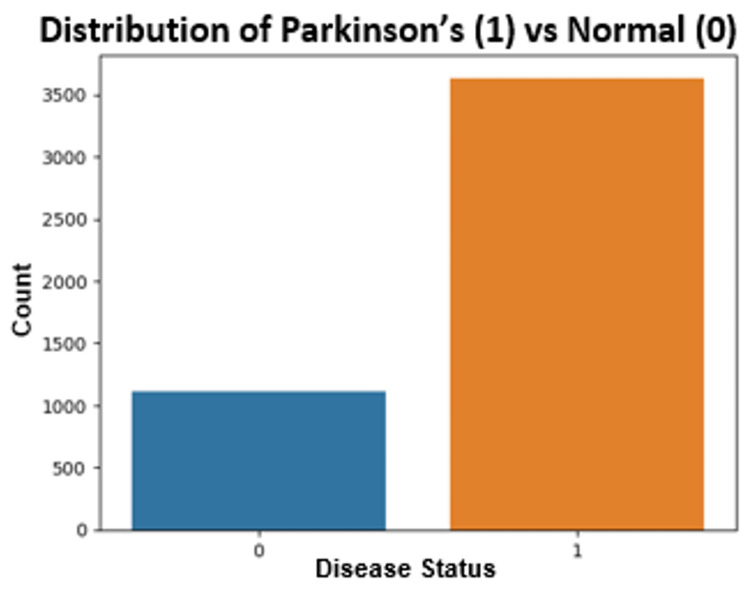
Number of data points from patients with Parkinson’s disease (PD) versus controls in the Tappy keystroke data. 0 represents control subjects, and 1 represents PD patients. "Count" represents the number of control subjects or PD patients.

The analysis revealed an accuracy of 72.79%, a precision of 76.50%, a recall (sensitivity) of 93.08%, and an F1 score of 83.97%. Figure 5 depicts the number of data points from patients with PD versus healthy controls.

**Figure 6:**
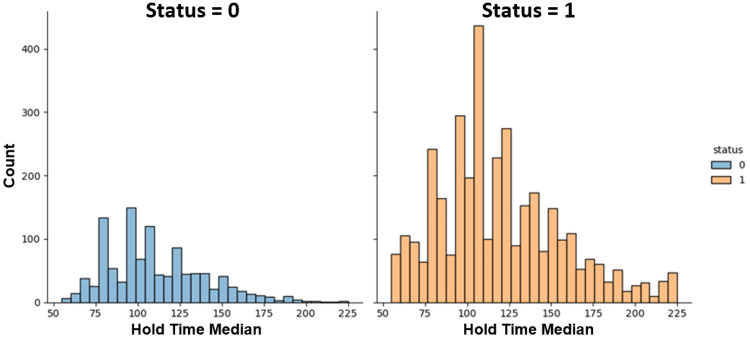
Distribution of hold time. 0 represents control subjects, and 1 represents Parkinson’s disease patients. "Count" represents the number of data points collected from the user. "Hold time" is the time between pressing and releasing each key.

Figure 6 depicts the distribution of hold time for healthy control subjects and PD patients in the Tappy keystroke dataset. The ML analysis shows a clear distinction between PD patients and healthy control subjects.

**Figure 7:**
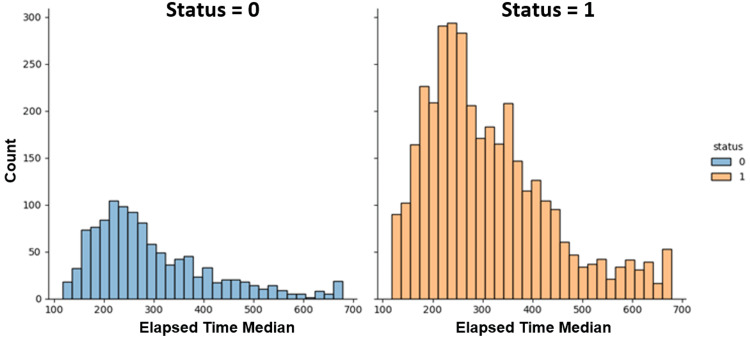
Distribution of elapsed time. 0 represents control subjects, and 1 represents Parkinson’s disease patients. “Count" represents the number of data points collected from the user. “Elapsed” time is the time (ms) between releasing a key and pressing the subsequent key.

Figure 7 depicts the distribution of elapsed time for healthy control subjects and PD patients in the Tappy keystroke dataset.

**Figure 8:**
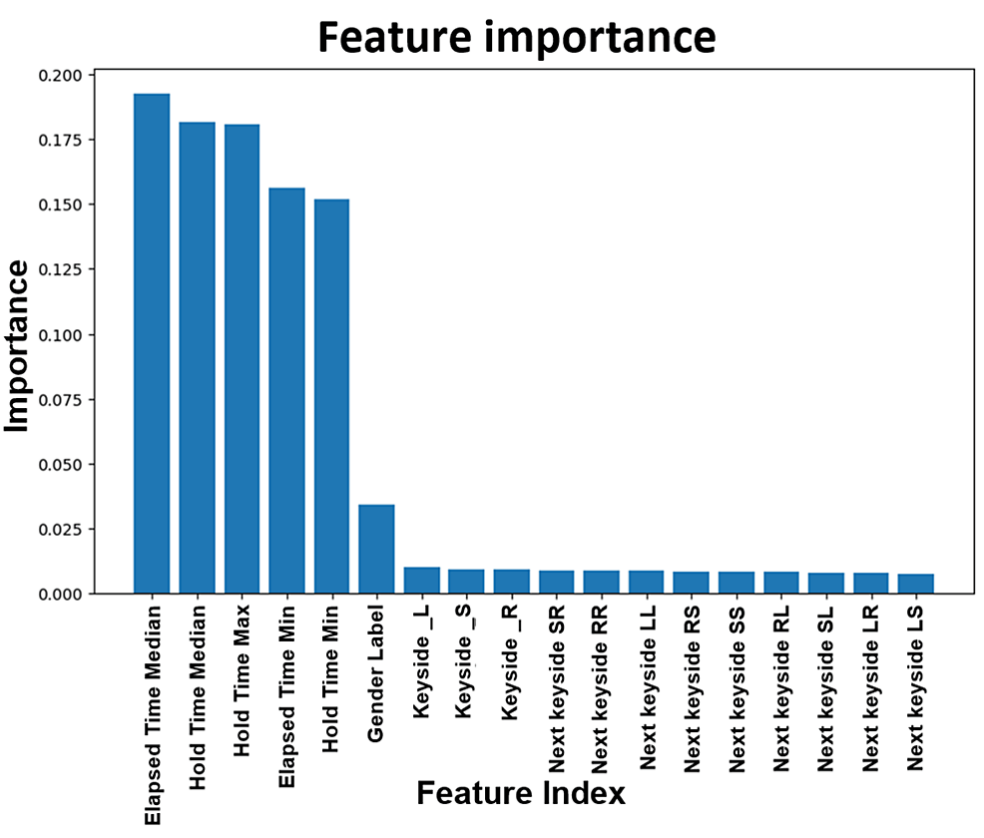
Feature importance excluding count in the Tappy keystroke data. "Hold time" is the time between pressing and releasing each key. "Elapsed time" is the time (ms) between releasing a key and pressing the subsequent key. "Keyside_L" represents keys pressed by the left hand. "Keyside_R" represents keys pressed by the right hand. "Keyside_S" represents the space key because it can be pressed by either the left hand or the right hand. "Next keyside" represents latency between successive keystrokes (elapsed time in ms from key down of one key until the next key down) categorized by same hand (LL or RR), opposite hand (LR and RL), and between the space key and left- or right-hand keys.

Figure 8 depicts the feature importance in the Tappy keystroke dataset analysis. When building a model based on Tappy keystroke data, important features to consider are the elapsed time median followed by the hold time median.

Spiral drawing data analysis

**Figure 9:**
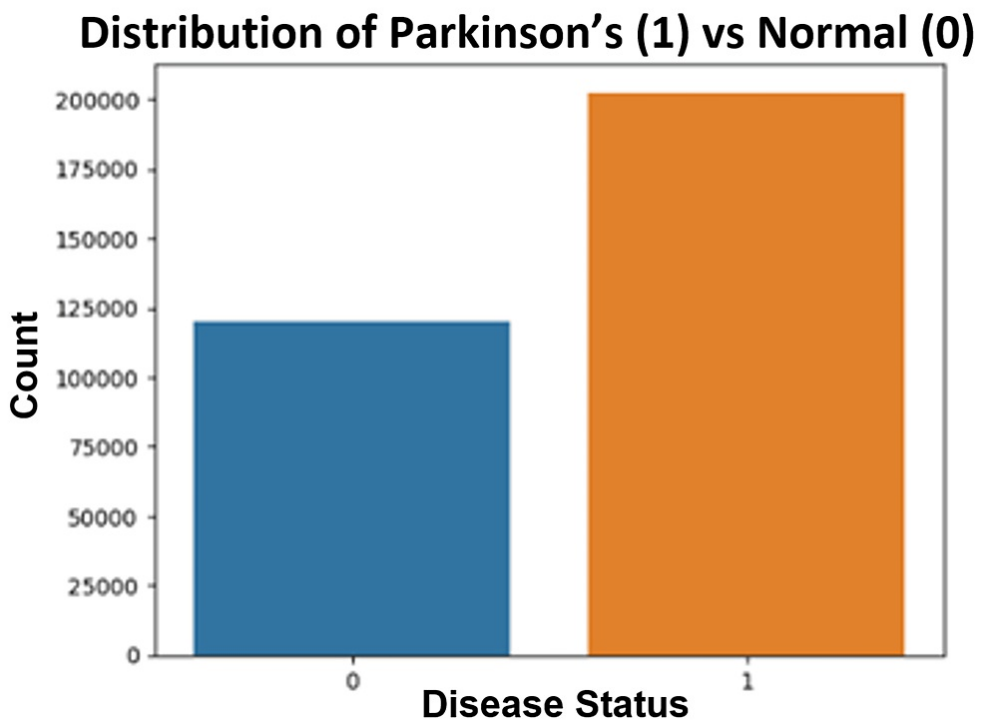
Distribution of data points between Parkinson's disease (PD) patients and normal controls in the spiral drawing dataset. 0 represents control subjects, and 1 represents PD patients. "Count" represents the number of control subjects or PD patients.

The data for the spiral drawing analysis was collected using the Static Spiral Test (subjects draw on the given spiral pattern), the Dynamic Spiral Test (the spiral pattern blinks at certain intervals, and subjects need to continue drawing), and the Circular Motion Test (subjects draw circles around a red point, see Figure 2). The ML analysis of the data revealed an accuracy of 70.97%, precision of 74.09%, recall of 82.89%, and an F1 score of 77.90%. Figure 9 depicts the distribution of data points between PD patients and normal controls.

**Figure 10:**
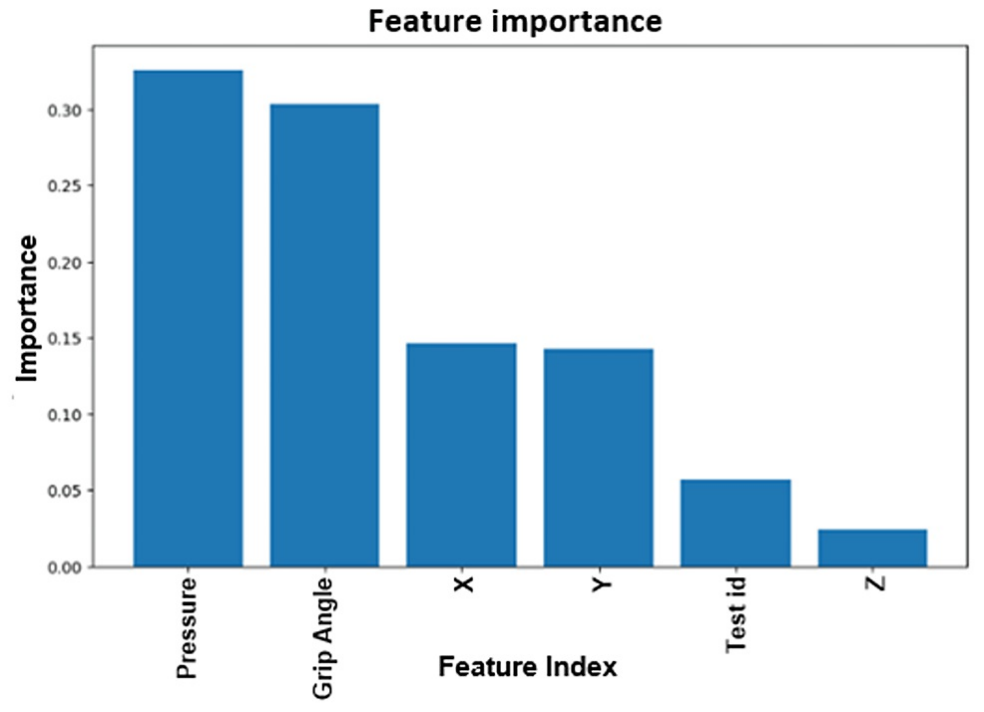
Feature importance between Parkinson's disease patients and healthy controls in the spiral drawing dataset. "Test ID" indicates which test was taken, i.e., Static Spiral Test, Dynamic Spiral Test, or Circular Motion Test.

Figure 10 depicts the feature importance between PD patients and healthy controls in the spiral drawing dataset analysis. The analysis considered X, Y, and Z coordinates, pressure applied to the screen, stylus grip angle, and timestamp (time consumed to complete the task). When building a model based on spiral drawings, important features to consider are pressure applied to the screen and stylus grip angle in the Dynamic Spiral Test.

Gait data analysis

**Figure 11:**
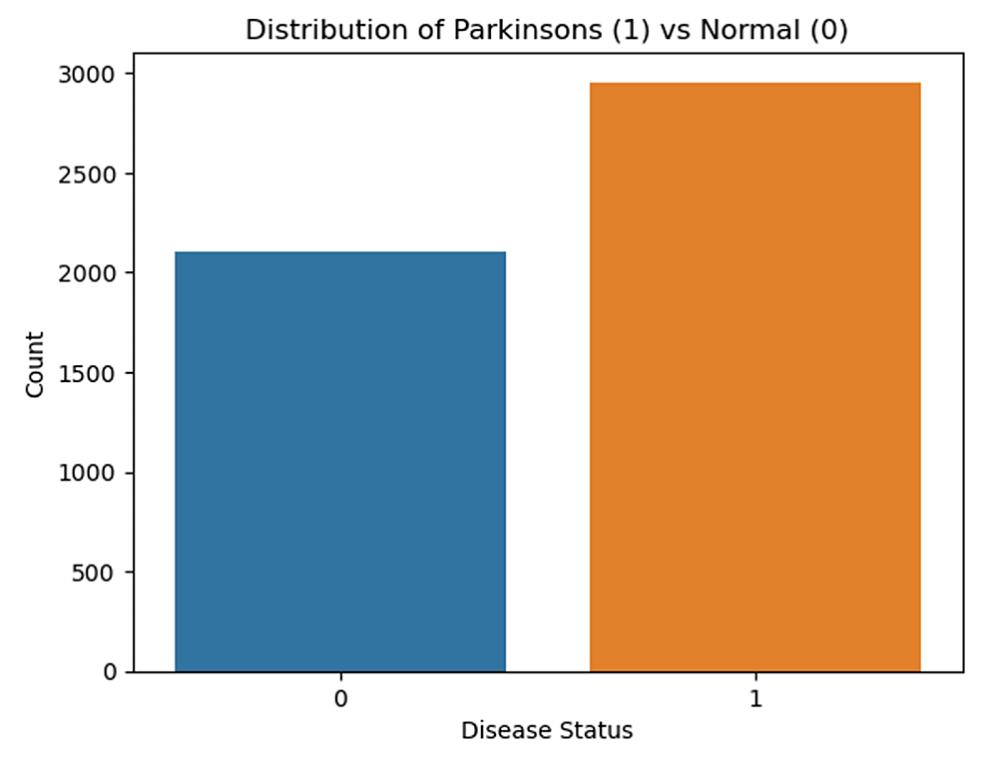
Distribution of data points between Parkinson's disease (PD) patients and normal control subjects in the gait dataset. 0 represents control subjects, and 1 represents PD patients. "Count" represents the number of control subjects or PD patients.

The analysis revealed an accuracy of 63.83%, a precision of 67.90%, a recall of 74.81%, and an F1 score of 70.01%. The dataset included time (in seconds), Vertical Ground Reaction Force (VGRF, in Newtons) on each of the eight sensors located under the left foot, VGRF on each of the eight sensors located under the right foot, total force under the left foot, and total force under the right foot. Figure 11 depicts the distribution of data points between PD patients and normal control subjects in the gait dataset.

**Figure 12:**
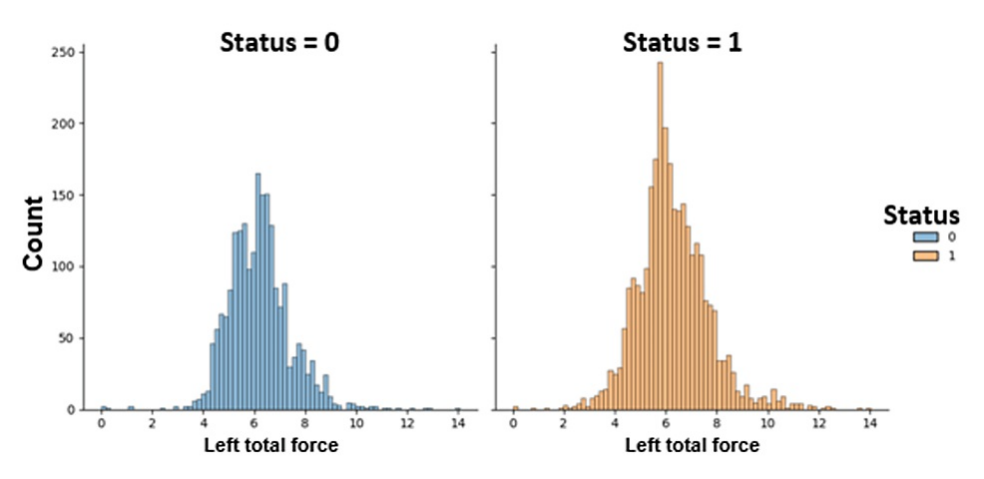
Distribution of the left total force for Parkinson's disease (PD) patients and non-PD healthy controls in the gait dataset. 0 represents control subjects, and 1 represents PD patients.

Figure 12 depicts the distribution of the left total force for PD patients and non-PD healthy controls in the gait dataset analysis.

**Figure 13:**
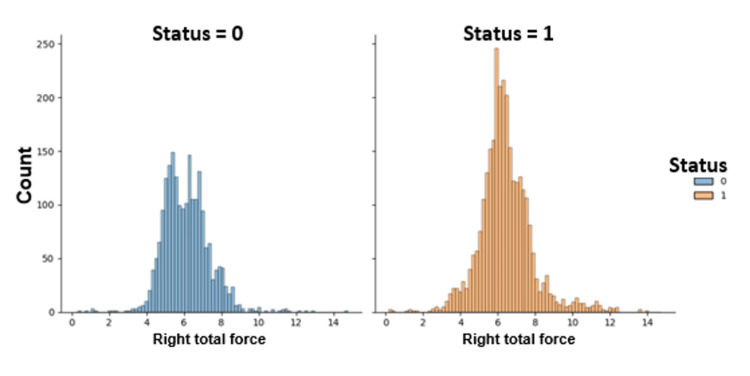
Distribution of the right total force for Parkinson's disease (PD) patients and non-PD healthy controls in the gait dataset. 0 represents control subjects, and 1 represents PD patients.

Figure 13 depicts the distribution of the right total force for PD patients and non-PD healthy controls in the gait dataset analysis.

**Figure 14:**
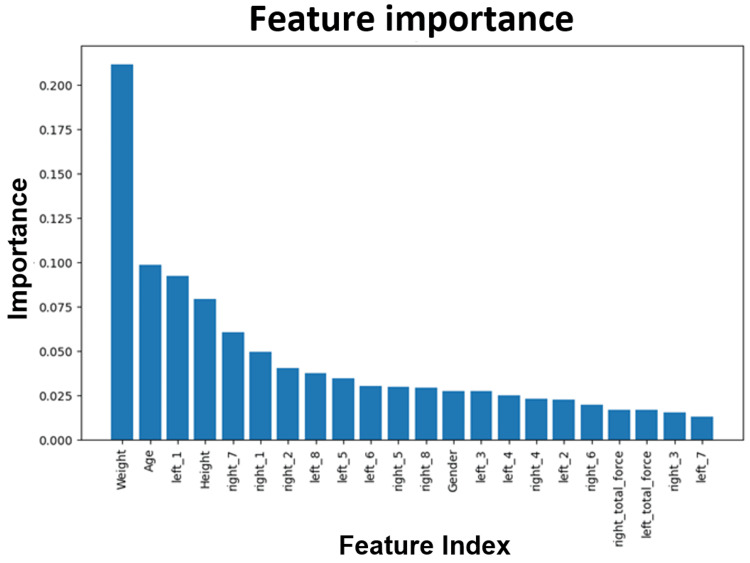
Plot of the feature importance between Parkinson's disease patients and healthy controls in the gait dataset. "Left" and "right" refer to different sensors on the left and right foot to measure applied force.

Figure 14 depicts a plot of the feature importance between PD patients and healthy controls in the gait dataset analysis. When building a model based on gait data, important features to consider are weight, height, and the difference in left and right force.

Voice analysis in patients with RBD

**Figure 15:**
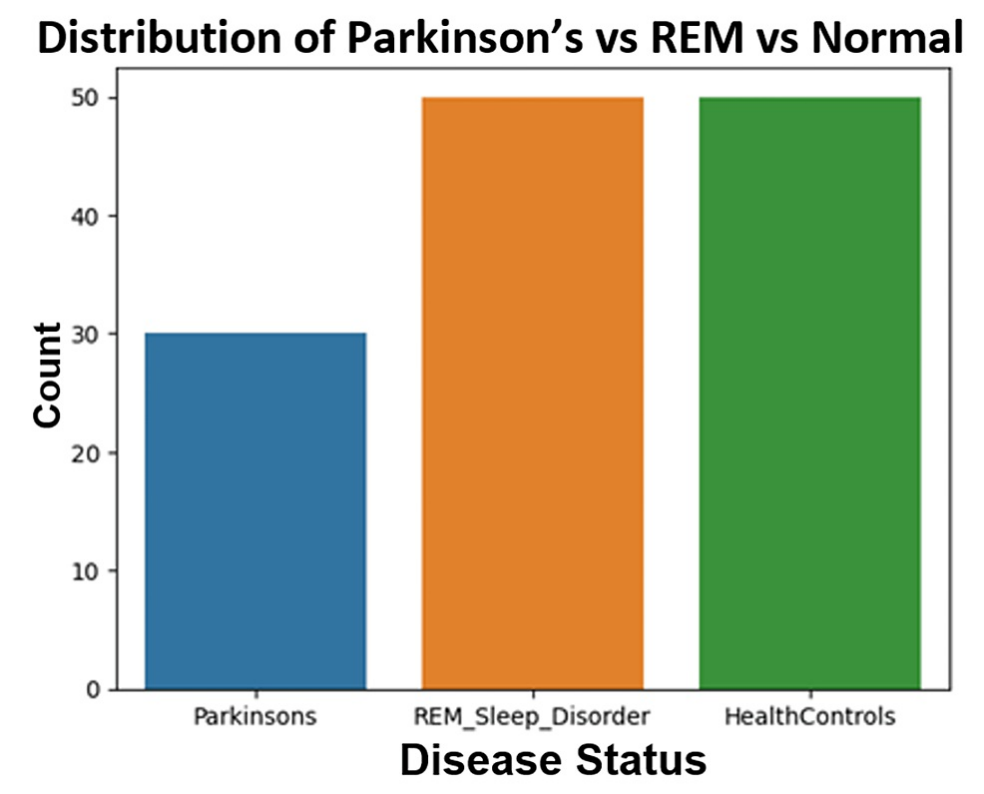
Distribution of Parkinson’s disease patients, RBD subjects, and healthy controls. RBD: REM (rapid eye movement) sleep behavior disorder.

Figure 15 shows the distribution of PD patients, RBD subjects, and healthy control subjects.

RBD vs. Normal Analysis

**Figure 16:**
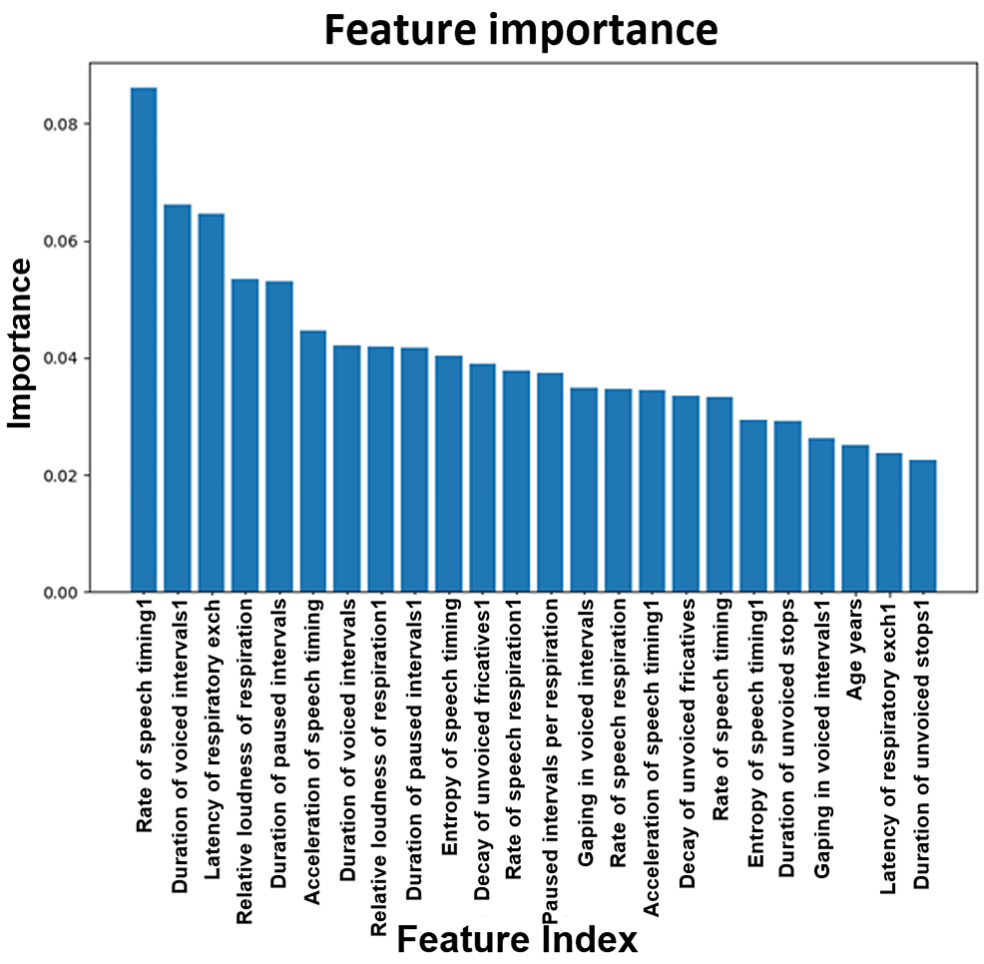
Plot of the feature importance for RBD vs. normal. RBD: REM (rapid eye movement) sleep behavior disorder.

Data was analyzed using a logistic ML model. Figure 16 shows a plot of the feature importance between RBD patients and healthy controls. When building a model based on RBD vs. healthy controls, important features to consider are the rate of speech timing followed by the duration of voiced intervals.

PD vs. Normal Analysis

**Figure 17:**
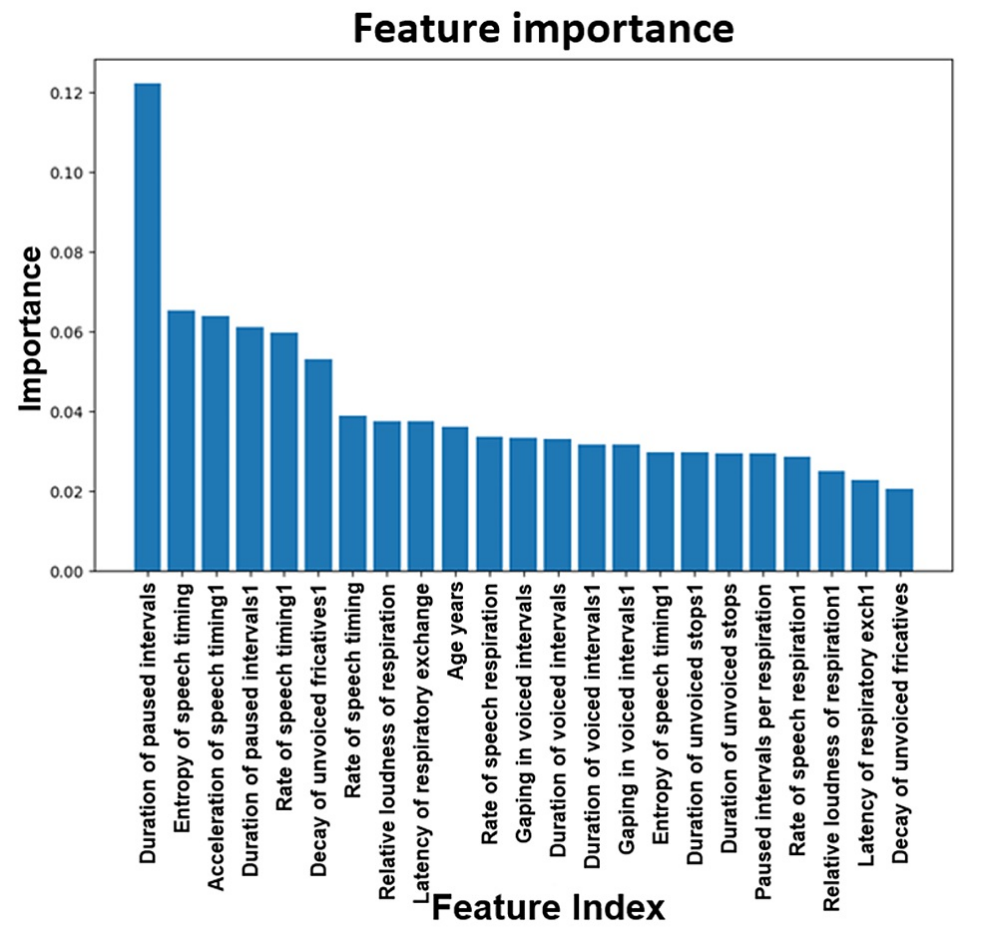
Plot of the feature importance for PD vs. normal. PD: Parkinson's disease.

Figure 17 shows a plot of the feature importance between PD patients and healthy controls. When building a model based on PD vs. normal controls in this dataset, important features to consider are the duration of pause intervals followed by the entropy of speech timing.

RBD vs. PD Analysis

**Figure 18:**
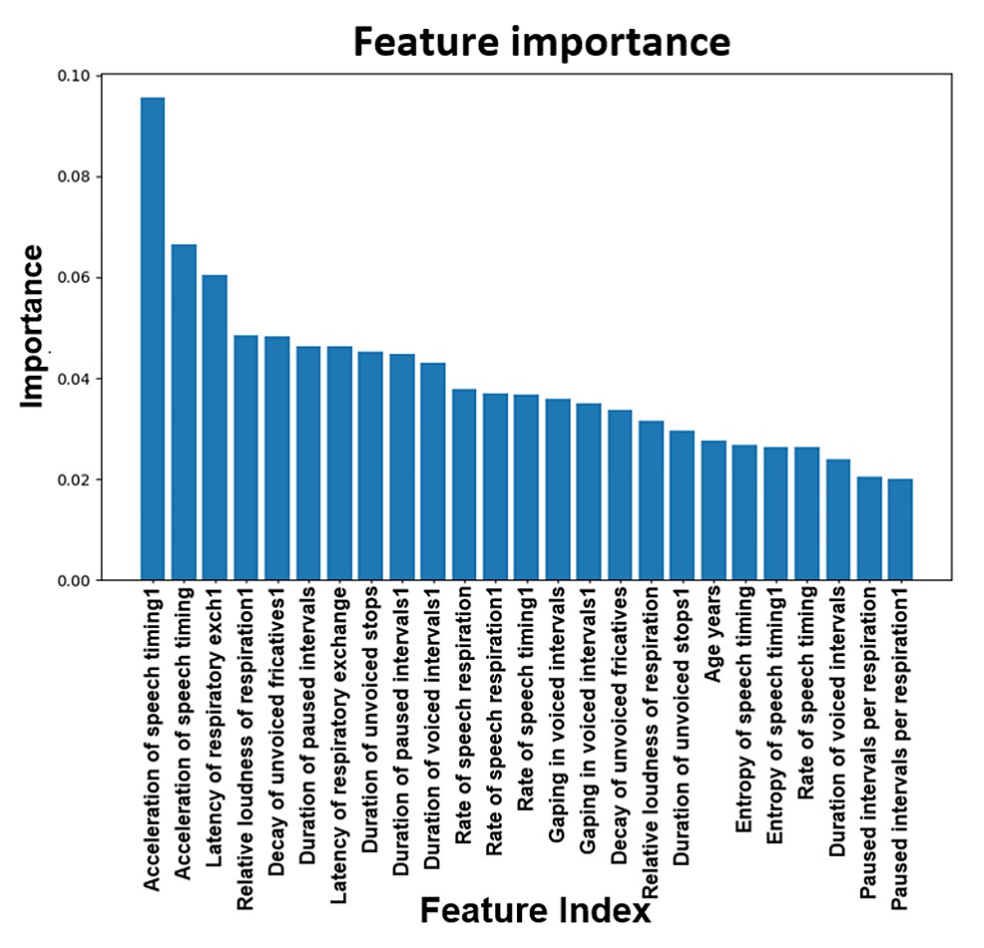
Plot of the feature importance for RBD vs. PD. RBD: REM (rapid eye movement) sleep behavior disorder; PD: Parkinson's disease.

Figure 18 shows a plot of the feature importance between RBD and PD patients. When building a model based on RBD and PD patients in this dataset, important features to consider are the acceleration of speech timing followed by the latency of respiratory exchange.

Table 1 shows a summary of the metrics for the results of the ML analysis. The results of these datasets demonstrate that voice is the best indicator for early PD.

**Table 1:** Summary of the metrics for results of ML analysis. RBD: REM (rapid eye movement) sleep behavior disorder; PD: Parkinson's disease; ML: Machine learning. * Logistic regression model for cross-validation; ‡ Data from the same study.

	Accuracy	Precision	Recall (sensitivity)	F1 score
Voice data	88.72%	90.86%	95.22%	92.77%
Tappy keystroke data	72.79%	76.50%	93.08%	83.97%
Spiral drawing data	70.97%	74.09%	82.89%	77.90%
Gait data	63.83%	67.90%	74.81%	70.01%
Voice data in RBD (vs Normal)*‡	70.00%	72.00%	70.00%	69.00%
Voice data in RBD (PD vs Normal)‡	66.00%	65.00%	66.00%	64.00%
Voice data in RBD (RBD vs PD)‡	55.00%	46.00%	55.00%	48.00%

## Discussion

The basal ganglia are involved in the planning, programming, and execution of motor tasks. Hence, speech, which is a complex motor skill, is very sensitive to disturbances in the basal ganglia, which is affected in PD [29,30].

Patients who act out their dreams during the REM phase of sleep, or have RBD, are at high risk (>80% likelihood) of developing PD [24]. Speech analysis in RBD patients was also carried out to see if the voice changes that occur in PD patients also occur in RBD patients. Speech is affected even in RBD patients, revealing that speech is not only a sensitive measure but also a very early prodromal sign of PD. Voice can be used as a clinical marker from the premotor phase to the development of motor symptoms. A non-invasive and easy-to-use method using voice samples in natural settings for the early and distinctive diagnosis of PD will be a valuable resource to help PD.

Analysis of voice, computer keystroke, spiral drawing, and gait in RBD patients can suggest which of these is affected the earliest and can be used as an early screening tool for PD. These parameters may also be used to monitor the progression of PD since affected patients are older and have trouble traveling for in-person visits. These tests can be self-administered, are inexpensive, non-invasive, can be performed by patients in their own homes, and data can be forwarded over the internet to the clinician’s office. These tests can be used to remotely monitor the effect of administered treatments [8,22].

An ML model based on the Random Forest algorithm was developed to analyze various PD characteristics that may be developing in patients before the clinical diagnosis of PD. The ML prediction model developed in this study may be helpful in current efforts to improve risk prediction in PD to help clinicians with early disease intervention and resource prioritization. The low accuracy of the analysis indicates that several PD-positive samples would be undetected and unclassified.

In the current analysis of voice, computer keystrokes, spiral drawings, and gait datasets, voice analysis is the best indicator for early PD diagnosis or prediction. Rather than using individual features, a combination of multiple features can be used to develop an ML strategy for more accurate early identification of PD. Combining all four features such as voice analysis, Tappy keystroke, spiral drawings, and gait analysis may improve accuracy. The current ML model should be improved by using larger datasets to improve prediction accuracy. Increased deposition of data into publicly available open datasets on sites such as Kaggle will increase the number of patients and controls available to improve the training of the ML models. Makarious et al. have shown that combining data modalities outperforms the single biomarker paradigm [1]. The ML model developed by Makarious et al. is useful for identifying large groups of individuals to monitor within health registries or biobanks [1]. However, the Makarious et al. model will flag 10 times more potential patients than will actually go on to develop the disease, while the current model is useful for real-time identification of people in the prodromal phase of PD [1]. The current model will be useful to detect the emergence of early symptoms in high-risk individuals.

The current data analysis was limited by different cohorts being used for different parameters and only limited datasets being available in the public domain of Kaggle. A limitation of this study is that constipation, smell, and RBD, which can be early predictors, were not included since this data could not be located on the publicly available Kaggle database. One shortcoming of combining different databases is that they are not from the same patients, so there is a need for more comprehensive multimodal data collection from the same PD patients and controls. Future studies should be designed to collect multiple parameters on the same patients prospectively from the prodromal to the clinical PD phase.

## Conclusions

Using non-invasive, home-based, inexpensive, and remotely accessed data from activities of daily living can help in the early diagnosis of Parkinson’s disease (PD). It can also help monitor the emergence of symptoms in patients at high risk of developing PD. For example, a background app installed on a smartphone can identify and record early aberrations in speech and flag them to the clinician. A background application installed on a computer can record variations in keystrokes and alert the clinical provider. Pressure sensors installed in shoes can continuously monitor changes in gait. These can be cost-effective ways to decrease the financial burden of PD over the coming decade.

The incidence of PD is increasing, and cost-effective methods are needed for early identification and timely intervention. This study is unique as it provides a machine learning approach to analyze early changes in multiple activities of daily living to identify the emergence of PD through remote analysis in real-time and cost-effective monitoring.
